# Using creative co-design to develop a decision support tool for people with malignant pleural effusion

**DOI:** 10.1186/s12911-020-01200-3

**Published:** 2020-08-05

**Authors:** Cheryl Grindell, Angela Tod, Remi Bec, Daniel Wolstenholme, Rahul Bhatnagar, Parthipan Sivakumar, Anna Morley, Jayne Holme, Judith Lyons, Maryam Ahmed, Susan Jackson, Deirdre Wallace, Farinaz Noorzad, Meera Kamalanathan, Liju Ahmed, Mathew Evison

**Affiliations:** 1 CLAHRC YH, Sheffield Teaching Hospitals NHS Foundation Trust, Glossop Road, Sheffield, S10 2JF UK; 2 School of Nursing and Midwifery, The University of Sheffield. Barber House Annex, 3a Clarkehouse Rd, Sheffield, S10 2LA UK; 3 Lab4living, Sheffield Hallam University, Cantor Building. Arundle Street, Sheffield, S1 2NU UK; 4 Academic Respiratory Unit, University of Bristol, Learning and Research Building, Southmead Hospital, Bristol, BS10 5NB UK; 5 Department of Thoracic Medicine, St Thomas’ Hospital, Guy’s and St Thomas’ NHS Trust, Westminster Bridge Road, London, SE1 7EH UK; 6 Pleural Service, Whythenshawe Hospital, Manchester University NHS Foundation Trust, Southmoor Road, Manchester, M23 9LT UK

**Keywords:** Creative co-design, Co-production, Malignant pleural effusion, Decision support tool, Complex intervention development

## Abstract

**Background:**

Malignant pleural effusion (MPE) is a common, serious problem predominantly seen in metastatic lung and breast cancer and malignant pleural mesothelioma. Recurrence of malignant pleural effusion is common, and symptoms significantly impair people’s daily lives. Numerous treatment options exist, yet choosing the most suitable depends on many factors and making decisions can be challenging in pressured, time-sensitive clinical environments. Clinicians identified a need to develop a decision support tool. This paper reports the process of co-producing an initial prototype tool.

**Methods:**

Creative co-design methods were used. Three pleural teams from three disparate clinical sites in the UK were involved. To overcome the geographical distance between sites and the ill-health of service users, novel distributed methods of creative co-design were used. Local workshops were designed and structured, including video clips of activities. These were run on each site with clinicians, patients and carers. A joint national workshop was then conducted with representatives from all stakeholder groups to consider the findings and outputs from local meetings.

The design team worked with participants to develop outputs, including patient timelines and personas. These were used as the basis to develop and test prototype ideas.

**Results:**

Key messages from the workshops informed prototype development. These messages were as follows. Understanding and managing the pleural effusion was the priority for patients, not their overall cancer journey. Preferred methods for receiving information were varied but visual and graphic approaches were favoured. The main influences on people’s decisions about their MPE treatment were personal aspects of their lives, for example, how active they are, what support they have at home.

The findings informed the development of a first prototype/service visualisation (a video representing a web-based support tool) to help people identify personal priorities and to guide shared treatment decisions.

**Conclusion:**

The creative design methods and distributed model used in this project overcame many of the barriers to traditional co-production methods such as power, language and time. They allowed specialist pleural teams and service users to work together to create a patient-facing decision support tool owned by those who will use it and ready for implementation and evaluation.

## Background

### Managing complexity in healthcare

Healthcare services are becoming increasingly complex. Improving these services or systems can be difficult as interactions between individual components are often multi-faceted; changing one individual part of these complex systems is unlikely to lead to meaningful change overall [1, 2]. There is an increasing need to involve patients and staff in the development of new interventions, to address the challenges complex problems and systems present, to make them relevant and applicable in practice [3–5].

This paper describes a service improvement project to design and develop a decision support tool: ‘my pleural effusion journey’. This tool aimed to address the complex problem of malignant pleural effusion (MPE) management. It involved patients and staff from three specialist pleural clinics from across the UK and used creative co-production as a means to achieve this. This paper does not set out to provide any formal findings from an evaluation of the tool. The purpose of this paper is to reflect on the potential contribution of creative co-production in the design and development of a complex intervention for an exemplar health care problem.

### Co-production and complex intervention development

Co-production has been growing in popularity over recent years as it is recognised that traditional linear approaches to generating and mobilising evidence do not always lead to changes in clinical practice or improved care [4, 5]. Co-production involves all stakeholders (service users and service providers) in the different stages of the research / service improvement process and takes into account local knowledge and context. It offers a more holistic and nuanced approach to address the evidence to practice gap than traditional research methods [1, 3, 6]. Knowledge in all its forms (research and experiential) is considered and blended to co-produce practical, contextually specific interventions that are owned by those who will use them and are more likely to be implementable in practice [7].

Co-production is a recognised method for complex intervention development [2] and a recently published taxonomy of approaches to developing interventions to improve health includes it in its partnership category [8]. In decision support intervention development specifically, it is now acknowledged that it is important to consider patients’ perspectives as well as the scientific evidence in content specification [9].

There is a lot of interest in co-production currently, but a lack of criticality as to what the term means. There are descriptions of the challenges of doing co-production such as power [3, 10, 11], language [10] and time [3, 11, 12], but no reports of practical attempts to address these. However, Langley et al. have proposed a framework that describes how creative methods address these challenges [7]. The creative co-production reported here is a practical response to the challenges drawing on the practice of the User Centred Healthcare Design and Translating Knowledge into Action themes of the National Institute for Health and Research Collaborations for Leadership in Applied Health Research and Care South Yorkshire (NIHR CLAHRC SY) and subsequently Yorkshire and Humber (YH).

**Fig. 1:**
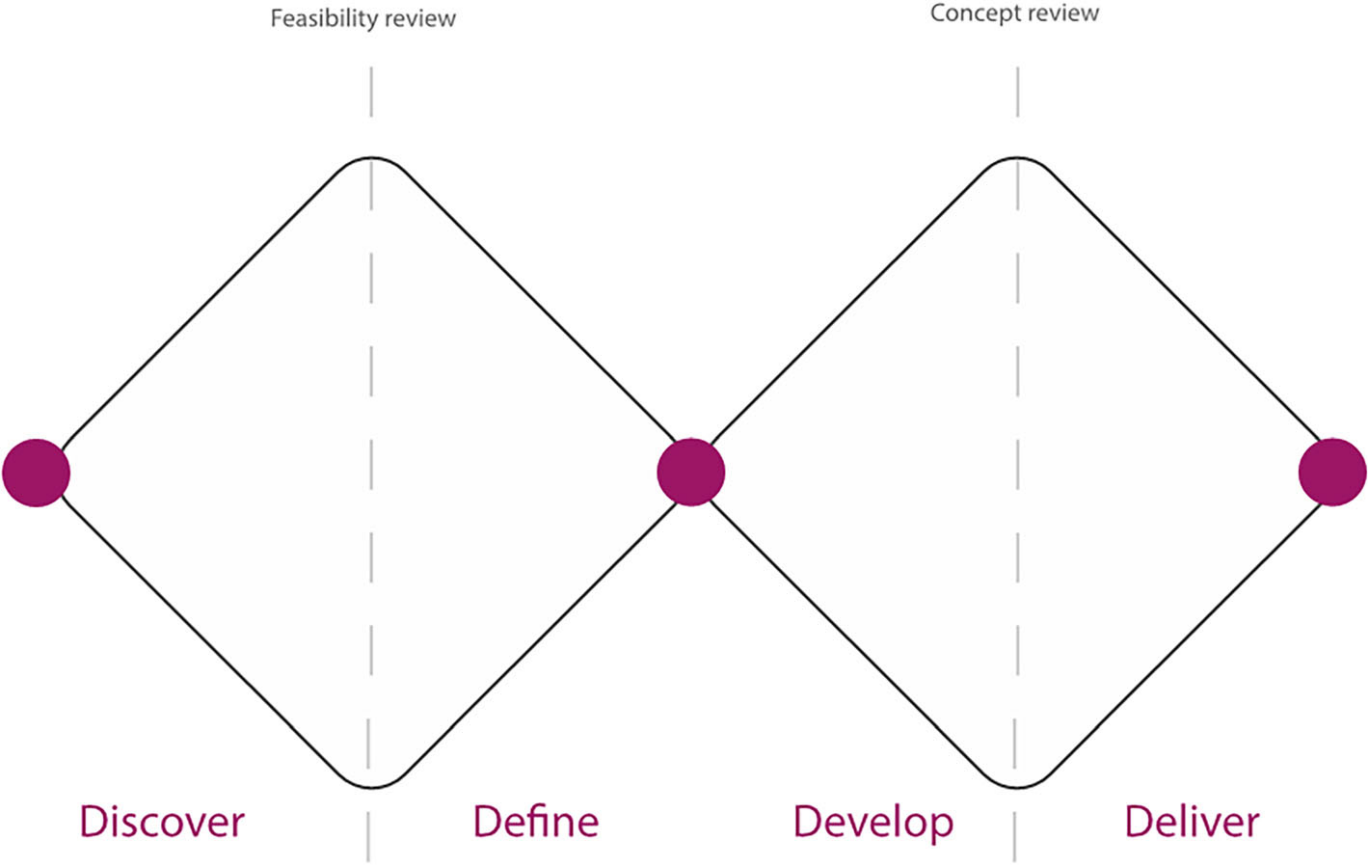
Better Services by Design 4 phase approach

A four phased process of creative co-production was adopted in the service improvement project presented here. The approach is based on the Better Services by Design approach [13]. This is a human-centred process of divergent and convergent thinking. It ensures all forms of knowledge are recognised and defined in the first stages. All ideas are considered in the latter stages before the best or most practical solutions are tested through an iterative prototyping process. The last phase consists of delivering a final prototype ready for evaluation and implementation [13] (Fig. 1).

Creative methods using visualisations and the making of design artefacts within the workshops formed the basis of our approach. These allow capturing the participant’s experience, knowledge, habits, behaviours and ideas and promote a shared common language that avoids professional jargon [7, 14]. The creative activities enable participants to unlock tacit knowledge and turn their ideas into real, visible and tangible objects that show their suggestions have been valued, listened to and acted upon [7, 10, 14].

The Translating Knowledge into Action (TK2A) theme of the NIHR CLAHRC YH has been using and developing this approach over the past 10 years. The TK2A team has a unique combination of clinical researchers and design researchers who have developed and now deliver this creative co-production approach. With both clinical and designer perspectives, the creative co-production approach is able to be flexible and responsive throughout the process and allows for the consideration of all aspects of the clinical service. Bringing together the clinical and designer perspectives which guide those of the participants ensures the methods fit the end goal whilst remaining focused on the clinical problems at hand.

This project aimed to develop a decision support tool to address the complex problem of malignant pleural effusion (MPE) management and it exemplifies the value of creative co-production methods.

### The complex clinical problem

Malignant pleural effusion (MPE) is an accumulation of fluid in the pleural space in the presence of malignant cells or tumour tissue. It is a common, serious problem predominantly seen in metastatic lung and breast cancer, and commonly in malignant pleural mesothelioma [15]. Recurrence of MPE is common and symptoms, which include breathlessness, pain, cough and reduced physical activity, significantly impair people’s daily lives [15]. Despite advances in the treatment of MPE, management remains palliative, focusing on relief from symptoms in order to improve quality of life. Prognosis is variable and multi factorial but on average life expectancy after diagnosis is between 3 and 12 months [15].

There are a number of different treatment strategies for malignant pleural effusion. There are clinical factors that mean in certain scenarios some of these interventions are not appropriate. In other scenarios all treatment options are possible. When fluid has been removed from the pleural space the key question is whether the underlying lung then re-expands to its normal size, allowing contact with the inside of the chest wall (‘expandable lung’).

If the lung is expandable then attempts at adhering the lung to the chest wall may be appropriate, with the aim of preventing re-accumulation of fluid in the future [16]. This procedure, called pleurodesis, can be achieved through a number of methods: injecting a liquid slurry of talc powder (which acts as a local irritant to cause inflammation and adhesion) through a temporary chest tube placed in the pleural space; directly spraying dry talc powder during a video-assisted operation; or through a longer term, tunnelled chest tube that remains in-situ for several weeks or months whilst the patient goes about their normal day-to-day activities at home [16].

No one intervention has been proven to be more effective in terms of pleurodesis success [17, 18]. However, from the subjective viewpoint of the patient, there are potential positive and negative sides to each approach; for example, the procedures involving talc require short inpatient stays.

If the lung does not expand (non-expansile lung or ‘trapped lung’) then pleurodesis will not succeed and the treatment options include repeated removal of fluid (pleural aspiration) or tunnelled chest tube described above, which is drained on a regular basis.

For all involved, this is a challenging area with a number of complex medical concepts to explain to the patient. Additionally, patients with a MPE tend to present with severe breathlessness, typically necessitating urgent, often same-day intervention. This puts great stress on the decision-making process which, for the patient would ideally include: understanding the concept of the effusion; the different treatment options; and which of these may be best suited to them at that particular time. Choosing which option is best for each patient depends on many factors [19]. It is in this setting that there is an unmet need for a decision support tool, one which can hopefully better support patients and clinicians to make the most appropriate decisions.

Three pleural teams from across the UK had already undertaken a body of work to explore the patient experience in this area, including an evidence review of MPE treatment options, and qualitative patient and carer interviews. This confirmed the need for better support for patients in order to make the right choice regarding their MPE treatment. Funding had been secured to develop a decision support tool following a successful application to an open call for applications from the North West Lung Centre Charity at Wythenshawe Hospital. However, further work was required to co-produce the content and format of the decision support tool before handing this to the software company to develop the final product. This creative co-production project aimed to undertake that co-production as a collaboration between the clinicians, patient and carers from three national centres and the TK2A team. Due to the distance and ill health of the service users, a novel distributed model of creative co-production was developed and used. This article considers the contribution of creative co-production techniques in developing a new intervention (in this case a decision support tool) to address a complex clinical situation.

### Aims

The aim of this distributed creative co-production project was to develop an initial prototype of a decision support tool for people with MPE, using participatory methods and a patient-led approach. Future studies will evaluate the clinical effectiveness of the tool.

## Methods

This was a distributed creative co-production project. Three clinical pleural teams were involved: St Thomas’ Hospital (London), Wythenshawe Hospital (Manchester), and Southmead Hospital (Bristol). Creative co-production workshops were conducted locally (distributed) with healthcare professionals, patients and their carers from the three sites, and supported virtually by the TK2A team. This was followed by a national workshop regrouping the core teams from the three sites. Prototype development meetings were held via teleconferencing to analyse and interpret insight generated by the workshops. The process was led by the TK2A team of the NIHR CLAHRC YH using their creative co-production approach. A designer was integral to the conduct of all stages, allowing developing of visual design artefacts to support the process as well designing the resultant prototype. As this project was classed as service improvement, NHS ethical approval was not required. However ethical principles were considered throughout the project and consent was obtained from all participants prior to their participation in the workshops.

### Aims of the creative co-production workshops

#### Distributed creative co-production workshop

To understand the lived experience of malignant pleural effusion and its management from both service user and provider perspectives to gain a shared understanding of the key issues to be addressed.

#### National creative co-production workshop

To develop ideas to support development of a MPE decision support tool.

### Sample

The distributed creative co-production workshops were attended by clinicians, patients and carers from each local site, as outlined in Table 1.

**Table 1:** Sample creative co-production workshops

Local distributed workshops	
Site 1	Local facilitator (Consultant physician × 3) 5 x Patients 2 x Carers Pleural physician Clinical nurse specialist
Site 2	Local facilitator (Physician Registrar) 5 x Patients 3 x Carers Consultant physician Clinical nurse specialist Clinical research nurse
Site 3	Local facilitator (Physician Registrar) 5 x Patients 4 x Carers Senior research nurse Student nurse
**National workshops**	
TK2A team	2 x Facilitators (design researcher and clinical researcher)
Clinical staff	2 x Consultants 3 x Registrars 3 x Nurses
Patients and carers	2 x Patients

The national creative co-production workshop participants were the 2 facilitators along with lay and clinical representatives from the local, distributed workshops.

### Data collection

#### Distributed creative co-production workshop

Due to the disparate locations and the ill health of the patient participants, a distributed model of creative co-production was adopted. This involved the TK2A team preparing the materials and providing PowerPoint instructions folded into a step-by-step video for the local facilitator on each site to conduct their creative co-production workshop. The local facilitator was provided with a detailed workshop schedule and copies of the resources to be used in written, audio and video format (Box 1). These were discussed prior to the workshop via teleconference with the three local leads. The resources were then refined and redistributed electronically to allow time for the local facilitators to print and familiarise themselves with the resources and activities they would be carrying out with participants.

**Box 1:** Schedule for the local distributed creative co-production workshop and national workshop

Workshop	Schedule
**Local, distributed workshops**	• PowerPoint presentation and video: welcome and introduction • Warm up exercise • Patient journey exercise • Discussion • Pleural effusion timeline exercise • Discussion • Pleural effusion experience exercise • Discussion • Pleural effusion information exercise • Discussion and close
**National workshop**	• Welcome and introduction • Warm up exercise • Feedback from local workshops • Discussion • Developing personae • Discussion • Working with personae to identify decision making journey and information needs • Discussion • Decision support tool content and format • Discussion and close

**Fig. 2:**
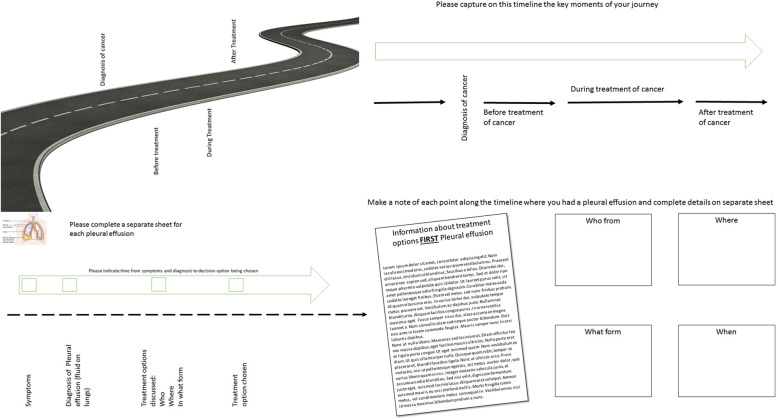
Distributed creative co-production workshop resources

The workshop facilitator took participants through a series of activities that considered their experiences of living with and managing MPE (Fig. 2). This allowed participants to gain a shared understanding of the challenges faced when giving and receiving information about MPE treatment options from both clinical staff and patient/carers perspectives. Each local workshop started with a standardised slideshow and introduction video which was pre-recorded by the TK2A team. Instructions for the initial warm up activity which encourage participants thinking creatively were also provided by a pre-recorded video. The local facilitators then followed the workshop plan and carried out the activities using printed versions of the resources provided.

Data was collected in the form of flip chart notes of discussions, completed workshop resources and field notes from facilitators. All these were sent to the TK2A team for analysis and discussion in the prototype development meetings.

### National creative co-production workshop

The national workshop took place 4 weeks later in London, with representatives from all clinical sites. Participants included a range of clinical staff, the TK2A team and two patients.

**Fig. 3:**
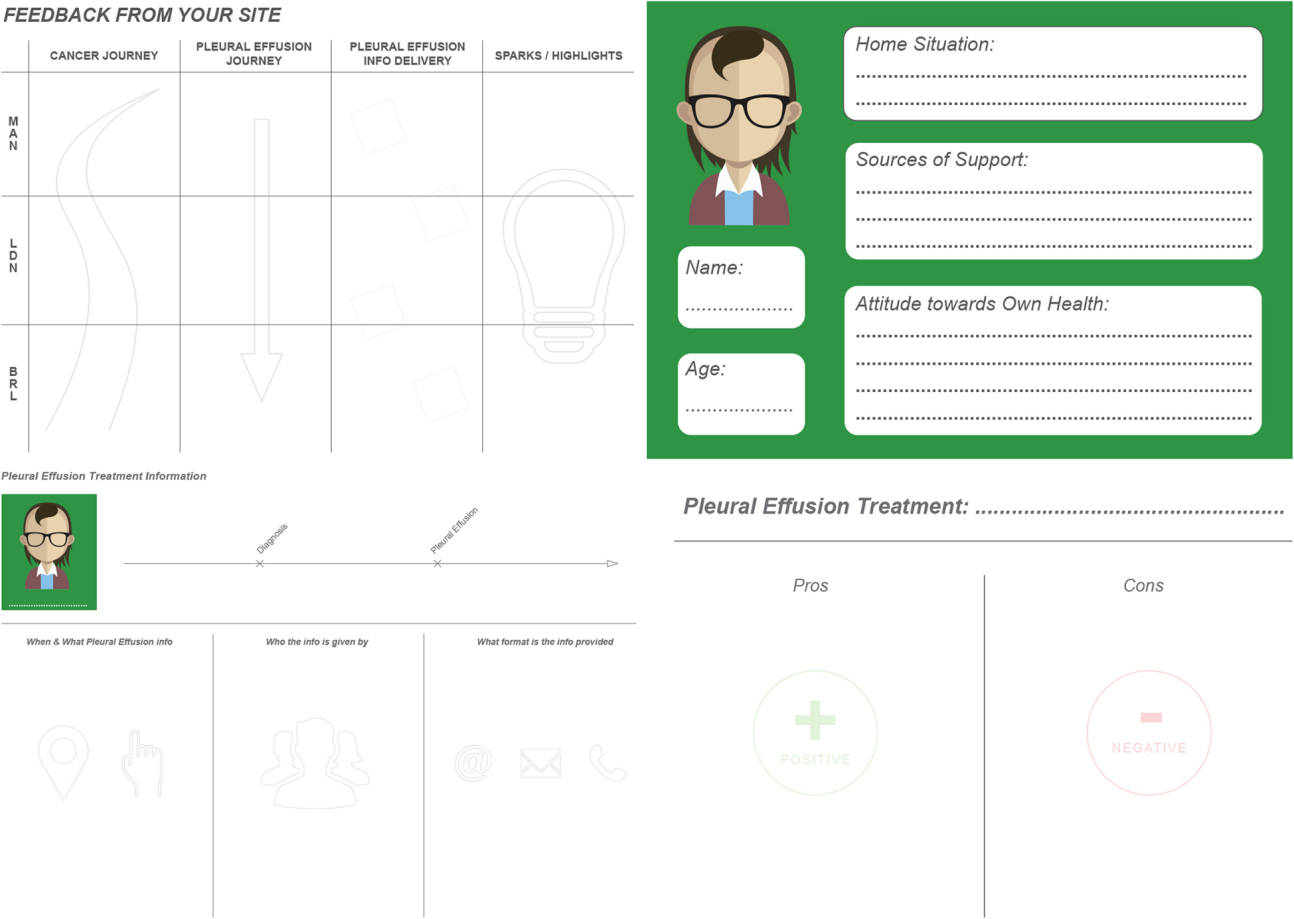
Creative co-design activities

In this co-production workshop, a series of creative activities were carried out by the participants, facilitated this time by the TK2A creative co-design experts. At the start of the national workshop each site was asked for their key insights from their local workshop in relation to the cancer journey, the MPE journey, MPE information delivery and sparks/highlights. This allowed the three teams to be able to see and discuss each other’s key workshop findings as well as verify the analysis that had been completed by the TK2A team remotely. The national workshop activities were supported by resources designed by the TK2A team (Fig. 3).

The national workshop enabled real time analysis and visualisation of ideas to occur by the design and clinical researchers present from the TK2A team. The creative activities enabled the participants to consider the findings from the first workshop and then explore the different treatment options and clinical and patient preferences. Personas were used to ensure the patient voice remained central to the process. The personas were developed by the workshop participants and were used to generate ideas about the decision support tool.

### Prototype development meetings

Prototype development meetings occurred at key stages throughout the process; these were:

- prior to the local distributed workshops
- following analysis of the data from the local workshops
- prior to the national workshop
- following analysis of the data from the local workshops
- following initial decision support tool prototype design to inform refinement
- at the end of the project to finalise the decision support tool prototype to send to the software company for development

Participants were the clinical lead for each site plus the TK2A team. All these meetings were conducted via teleconferencing. Notes on design discussions and decisions were recorded as data.

### Data analysis

Analysis of the data was iterative. It occurred throughout the process with findings informing the subsequent stages in the process of decision support tool development.

The local facilitators at each of the distributed workshops sent their completed resources back to the TK2A team electronically. The data was analysed after the workshops by the clinical and design researchers who had not been present at the workshops. Key themes and trends were identified by the TK2A team from the data separately for each of the three sites. These findings were discussed in a prototype development meeting to generate consensus regarding their meaning. The implications of the data for the decision support tool were considered. Resources were then developed for the national workshop based on these findings.

Analysis of activities in the national workshop used the same approach as per local meetings.

Further analysis of the final ideas from the national workshop was carried out by the TK2A team post workshop.

### Findings

The key experience-based findings from the workshops were summarised as follows:

### The distributed creative co-production local workshops

1. Patients were more concerned about the management of the symptoms of MPE as an immediate priority rather than their overall cancer treatment. This was due to the life limiting symptoms MPE causes.

2. People with MPE would prefer to receive information regarding treatment options in a timely manner, preferably by a specialist pleural team, and in a variety of formats including verbal, written and animation. However, visual information was of key importance to facilitate understanding of their MPE and therefore decision making. If advice from a specialist pleural team were not possible, then information should be provided in a consistent manner from other health professionals (e.g. physicians, oncologists, nurses).

3. If sign-posting was to occur, it needed to come from a reliable source (e.g. cancer charity websites like Macmillan or Mesothelioma UK).

4. Any information resource that supported treatment decisions needed to be available to people with MPE and their carers at key moments throughout their cancer journey.

Positive feedback was received regarding the distributed workshops themselves. One workshop participant (a student nurse) described the process of being able to hear the perspective of someone living with MPE as ‘*the best learning experience of their training to date*‘.

### The joint national workshop

The findings from the joint national workshop provided vital understanding of what the content and format of the decision support tool needed to be. The workshop findings indicated that the main influences on people’s decisions about their MPE treatment were:

1. Personal aspects of their lives (e.g. how active they are, what support is available at home);
2. Emotional and practical support such as support regarding worries, concerns and fears about treatment options.
3. Perceptions of underlying health, and ability to endure treatments and pain.

These were embodied in the prototype tool by prompt questions.

A clear message was that there is no ‘one size fits all’ solution for MPE management. Therefore, these factors that would facilitate access or appropriateness of different treatment options were made part of the prototype tool. Participants agreed that the decision support tool itself needed to be available in different formats to ensure its accessibility for a range of patients, regardless of their age, level of social support or distance from the hospital - factors identified as important in the workshops. A website platform seemed to be the most acceptable and accessible format. However, this was conditional on supporting information being available supplied by healthcare staff in the clinical environment.

Consensus was reached regarding the key content for the web-based decision support tool (Box 2). Recommended content included information about what a pleural effusion is, its cause, treatment options and implications.

**Box 2:** Core content for a MPE decision support tool

1) What is a pleural effusion? 2) Why it happens and what causes it. 3) General terminology and goals of treatment. 4) Details of the different treatment options. 5) What’s important to me (the person with MPE). 6) Further support in the form of trouble shooting, patient stories and links to other resources.	

The TK2A team used these findings to develop a first prototype in the form of a video representation of an online decision support aid. This showed that:

1. People with MPE could access the decision support tool from home via a tablet or laptop computer prior to coming to their hospital appointment. It could therefore be used by people in preparation for a clinic appointment, and in associated discussion of treatment options.
2. It could also be used as a support tool within a healthcare appointment. The latter was important if the tool was going to be used by people who could not use technology themselves due to illness, disability or preference.

**Fig. 4:**
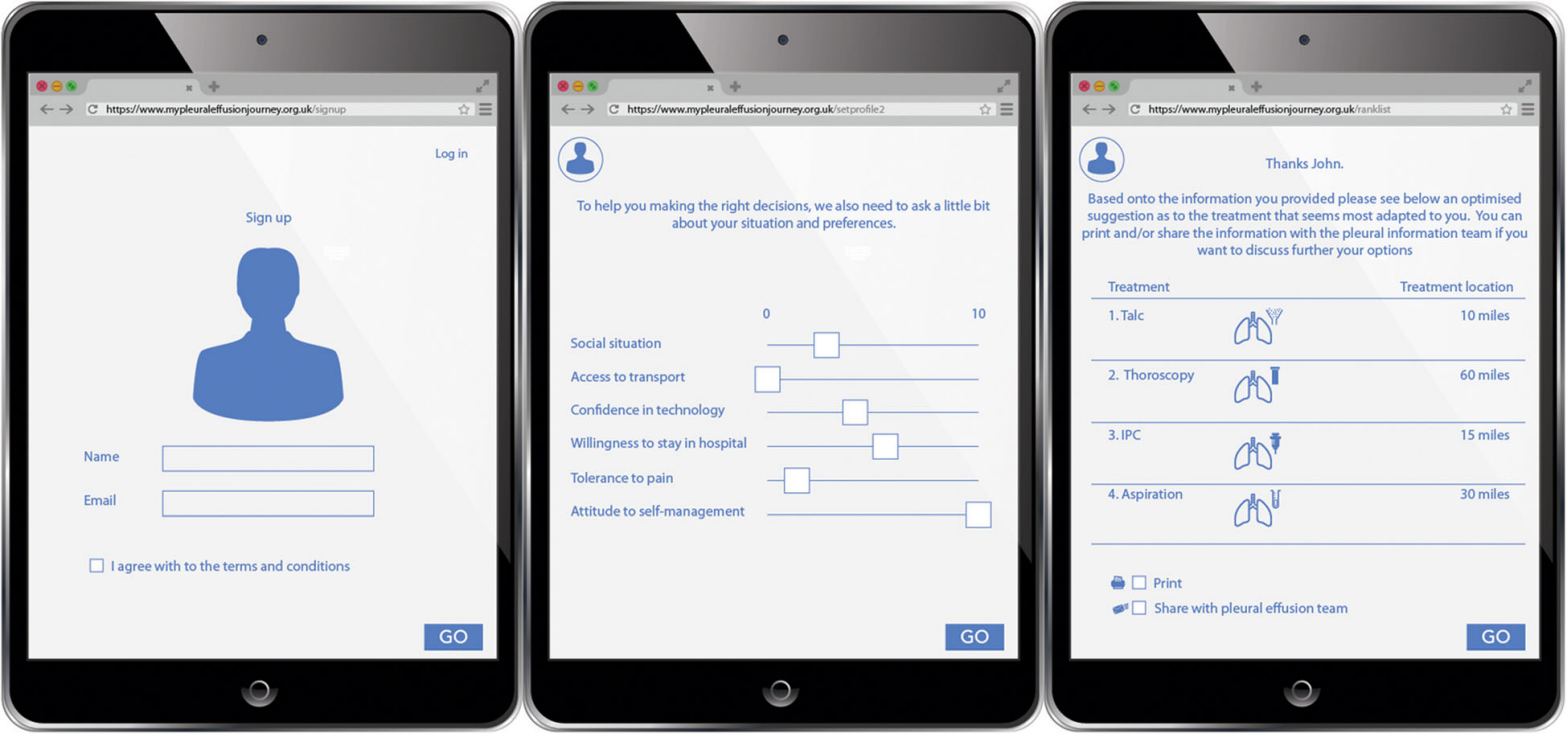
Prototype 1

The developed tool gives patients and carers information regarding the treatment options and helps them decide the most suitable option for them. It also takes into consideration the key influencing factors identified through the creative co-production process. These factors related to personal circumstances such as their tolerance to pain, how close they live to the hospital and whether they were happy to self- manage. The video was sent to the three participating pleural teams who were invited to give initial feedback prior to a teleconference to further discuss it in more depth. To facilitate remote conversation, slides of the website were produced (Fig. 4).

**Fig. 5:**
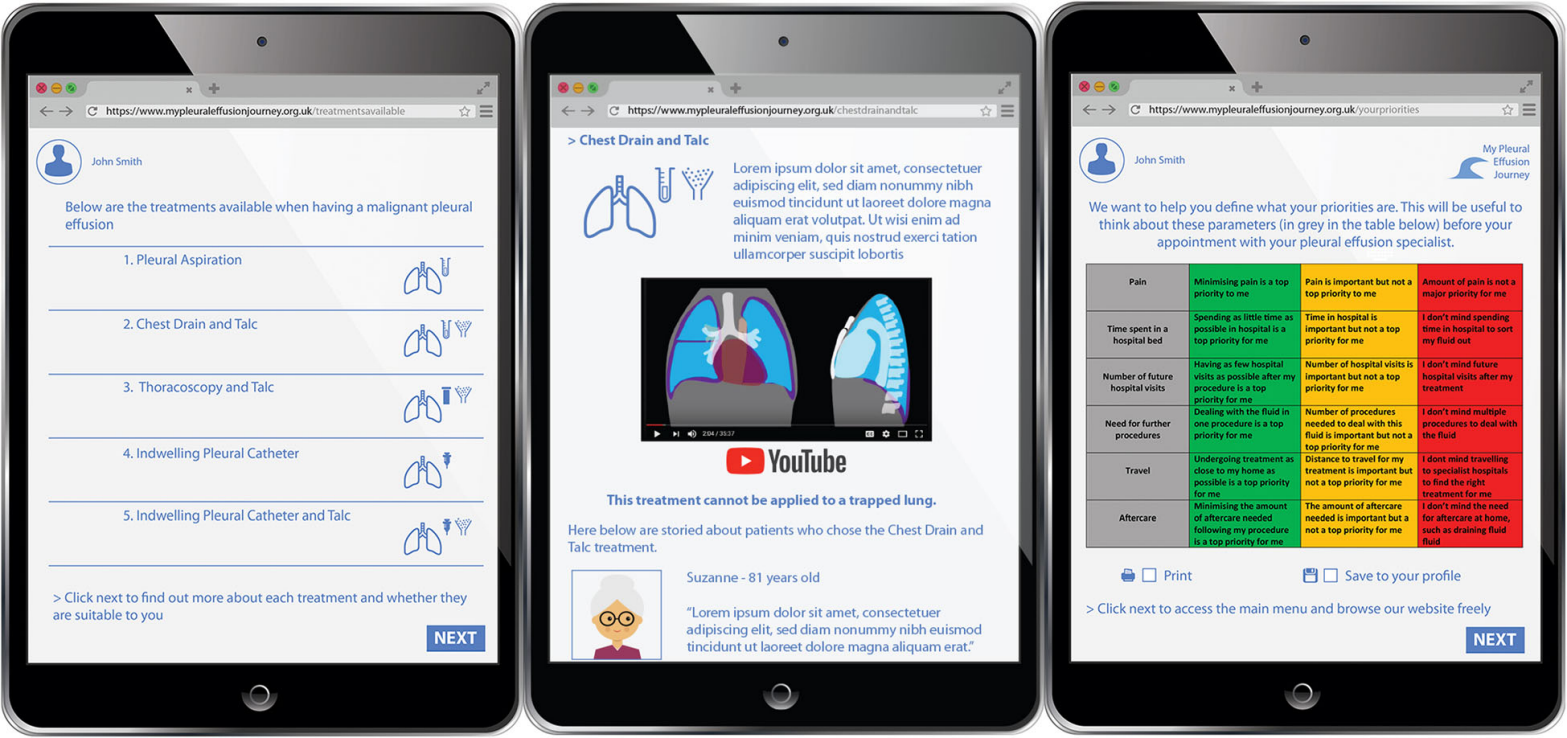
Prototype 2

Prototype 1 received positive feedback. However, there were concerns that the tool content could be interpreted as prioritising one treatment over another without allowing for discussion with a consultant.. Following this feedback, a second iteration of the video prototype was developed by the TK2A team incorporating a traffic light system (favoured by some of the clinicians in the workshops) which allowed the patient to see which treatments were available to them based on their personal circumstances (Fig. 5).

Further teleconference discussion took place between the TK2A team and the three pleural teams. Once the core structure and the visual representation were refined, the pleural teams were tasked with writing the clinical content and gathering patient stories. The content along with the video representation was then passed on to a software company who have developed a web based version of the tool ready for testing. This can be found at https://mypleuraleffusionjourney.com [20].

## Discussion

It is known that the implementation of evidence, into clinical practice is challenging [3, 5]. It is recognised that involving service users and providers in the evidence generation and intervention development process can help to overcome some of the problems faced compared to more traditional linear methods [3–5]. Co-production is growing in popularity as a way to engage service users and providers in applied health care research to help bridge the knowledge to practice gap [3–5] and help reduce research waste [8]. However, it is used in many different ways and not always described adequately in the literature [21]. This project describes a distributed form of co-production using creative co-design methods to address the challenge of co-creating a practical, contextually specific decision support tool for the management of MPE.

### Creative co-design

The creative and prototype development activities adopted in this project proved successful in co-creating a prototype complex intervention. The process overcame some of the common barriers to co-production in healthcare, namely, power, language and time. They also enabled a participatory approach to content specification in decision tool design and development as recommended by Elwyn et al. [9]

The participants in this project engaged in ‘making’ through creative activities in order to explore and reflect on their experiences; this helped to address to some extent the barriers of power and language. The creative activities enabled them to share and express themselves in an inclusive environment using a common language [7, 10]. It has been proposed that co-production activities that promote inclusivity and the development of meaningful, egalitarian partnerships between participants, unlocking tacit knowledge and encouraging different ways of thinking, can lead to more useful and implementable solutions [6, 7, 21].

The prototype decision support tool took the form of a visual representation of a web-based intervention but in video format. The short video included commentary through each screen allowing participants to get a feel for what the resource could be like and contain without having to wade through pages of descriptive text. This allowed everyone involved, regardless of profession or literacy, to be able to understand the concept of the proposed decision support tool. This visual technique is one that is recommended and often deployed in the creative design phase of web-based decision tool development [9].

Basing the co-production process on design principles encouraged a solution-focused approach and gave participants the permission to think beyond the usual constraints of their working environment. Having a design facilitator enabled visualisation of thoughts and ideas as they arose. This allowed real time synthesis of occurring knowledge, for example through drawings, which was presented in a form that was easy to understand and which accurately represented participant’s views [7].

Design prototyping is still not commonly used in a health care context, although it is starting to gain attention [5–7]. Prototyping in this study turned participants’ ideas into something tangible that helped generate more useful and practical feedback to drive the idea forward [6, 7]. Expense is always a factor when developing and designing interventions in health care. This study demonstrated that initial prototypes that aim to generate useful feedback do not need to be highly polished, expensive products [7]. When prototyping, Bec et al. argue that designers constantly have to make compromises about the level of fidelity of the prototype to make based on the available resources (e.g. time, money) and the type of feedback sought [22]. In this study, the use of low fidelity prototypes, that are quick, easy and cheap to make, allowed iterative cycles of feedback and development to occur. This co- created, visible and tangible object (a video representation in this case) is a physical embodiment of participants’ ideas and demonstrates that their suggestions have been valued, listened to and acted upon and therefore give them a sense of ownership [6, 7, 10]. This process of creative co-production from which the prototypes emerged, an online decision support tool in this case, mean that the final product is more likely to be implementable [7].

### Distributed model of creative co-production

Using a distributed creative co-design approach enabled the project to bring together three disparate teams and enabled the voices of all stakeholders to be heard. Hence, barriers such as time, geographical distance and poor health of service users could be overcome. The initial distributed workshops ensured the lived experiences of all stakeholders (service users and providers) were explored allowing a synthesis of evidence (experiential and research) to occur to inform the content specification phase of the decision tool development. Different perspectives were shared through the creative resources prepared by the specialist TK2A team. It might be argued that other methods of co-production could have delivered similar results. Examples include experience-based co-design which has an online toolkit of resources for local facilitators to use to deliver their own co- design workshops [23]. However, the benefits of having workshop activities and resources that are designed within the project, bespoke to the participants’ experiences proved successful here. These were prepared by design and clinical researchers with expertise in the use of the creative co-production techniques, following consultation with the specialist sites, with clear instructions that were written, verbal and visual (via paper, PowerPoint and video), which enabled more meaningful and engaging activities to be developed. This also saved busy clinicians (who acted as local facilitators) invaluable time as they did not have to plan and prepare the workshop themselves.

In this project the distributed creative co-production workshops were facilitated by local leads but planned and designed by design and clinical researchers. The level of involvement of the designer was significant and embedded at every stage. This genuine collaboration with designers allowed techniques such as drawing and making to be adopted as and when they were most fruitful, not just in the creative design phase of decision tool development as suggested by Elwyn et al. [9].

The distributed model of local and national workshops and the prototype development meetings enabled everyone that had been involved in the creative co-production process to remain involved throughout the project. The prototype could be viewed and feedback reviewed electronically (and via the prototype development teleconference meetings) without physically having to bring the three disparate groups of participants back together again in one place. It is unlikely that the feedback and subsequent prototype iterations would have developed in the same way without the sustained engagement of the participants from the three sites. Sustained inclusion in co-production is recognised as being difficult [21]. The distributed model of creative co-production successfully overcame some of the difficulties often experienced with recruitment, retention and sustained involvement of participants in the co-production process. It also saved valuable clinical time for the health professionals involved.

### Limitations of the study

This study describes the creative co-design methods used to develop a prototype decision support tool for the management of MPE only. The effectiveness of the intervention is not certain as no evaluation of the prototype in the clinical setting has yet been undertaken. However, the decision support tool has now been developed by a software company and funding is being sought to evaluate the prototype tool.

One limitation of the project was the small number of patients who had input across the study process. This was due to their ill health, which meant their input diminished beyond the first local workshops. However, this was pre-empted and addressed to some degree by the use of personas to represent the characteristics of a broad range of MPE patients to ensure their needs were considered throughout the creative co-production process. The personas used in the national workshop and throughout the prototype development were grounded in the patient experiences shared in the local workshops.

Co-production techniques are often criticised for their lack of generalisability. The findings from the workshops and the subsequent developed prototype decision tool were never intended to be generalisable beyond the local context within which they were co-created. That said, this was a national service improvement project involving three separate pleural teams in the UK; hence it could be argued, due to the multi-site involvement, the resultant prototype is more generalizable.

Finally, creative co-production is a resource intensive approach. The distributed model used in this project provided an efficient approach to prototype development and reduced the impact of participation to some degree on patient and clinician time.

### Implications for future co-production of complex interventions

The creative co-design methods used in this project attend to the development stage of the complex intervention development process [2] and fit both within the recent taxonomy of recommended approaches [8], and the process map for decision tool design and development recommended by Elwyn et al. [9]. It is recognised that involving appropriate stakeholders throughout the complex intervention development process, taking account of context and considering practicalities of implementation early on in the process, is likely to lead to more relevant and successful change in practice [2, 8]. However, there are many practical barriers in projects requiring such an inclusive approach. The distributed creative co-production approach could provide a useful way of overcoming those obstacles.

This project involved clinical and design researchers working together with all relevant stakeholders. This led not only to a blurring of traditional academic and practice boundaries, but also, through the addition of the practical and pragmatic contribution of design, to the engagement of users and carers in the creation of a complex intervention. This shared understanding and knowledge was used to co-create a practical and contextually specific solution to the complex problem of MPE management ultimately owned by those that created it. This approach therefore has the potential to address, understand and overcome common implementation challenges.

The distributed method used in this project has implications for future use of co-production in health care. It enabled multi-site involvement and a more flexible approach in terms of workshop planning, facilitation and prototype development.

## Conclusion

Creative co-production and the distributed method used in this service improvement project have many strengths compared to more traditional approaches to knowledge synthesis and complex intervention development. The approach attends to the challenges of power, language, and time which are recognised barriers to the achievement of successful change in healthcare as well as fitting within the suggested process map for decision tool design and development. Some might argue that this approach does not fulfil the needs of academic rigour or produce generalizable solutions. Nonetheless, the attention to context and the production of a prototype informed by the needs of those who will use it leads to more practical, fit for purpose interventions that are more likely to be usable in practice. Further research into both the distributed and creative co-production approaches is therefore warranted to critically examine their merit in complex intervention development and service improvement.

## Data Availability

CLAHRC YH finished in September 2019. The data used for this case study is therefore no longer available.

## Abbreviations

NIHR: National Institute of Health Research
CLAHRC SY: Collaboration for Leadership in Applied Health Research and Care South Yorkshire
CLAHRC YH: Collaboration for Leadership in Applied Health Research and Care Yorkshire and Humber
TK2A: Translating Knowledge into Action
MPE: Malignant pleural effusion

## References

[1] Reed JE, Howe C, Doyle C, Bell D. Simple rules for evidence translation in complex systems: a qualitative study. BMC Med. 2018;16(92):1076–9. 10.1186/s12916-018-1076-9 PMC6009041 29921274

[2] Craig P, Dieppe P, Macintyre S, Michie S, Nazareth I, Petticrew M (2008). Developing and evaluating complex interventions : new guidance. Br Med J.

[3] Greenhalgh T, Jackson C, Shaw S, Janamian T (2016). Achieving research impact through co-creation in community-based health services: literature review and case study. Milbank Q.

[4] Green LW (2009). Making research relevant: if it is an evidence-based practice, where’s the practice-based evidence?. Fam Pract.

[5] Holmes BJ, Kelly MP, Marshall M. Mobilising knowledge in complex health systems: a call to action. Evid Policy. 2017;13(3):539–60.

[6] Robert G, Macdonald AS (2017). Co-design, organisational creativity and quality improvement in the healthcare sector: ‘designerly’ or ‘design-like’?. Design for Service: Key Issues and New Directions. Bloomsbury Academic.

[7] Langley J, Wolstenholme D, Cooke J (2018). ‘Collective making’ as knowledge mobilisation : the contribution of participatory design in the co-creation of knowledge in healthcare. BMC Health Serv Res.

[8] O’Cathain A, Croot L, Sworn K, Duncan E, Rousseau N, Turner K (2019). Taxonomy of approaches to developing interventions to improve health: a systematic methods overview. Pilot Feasibility Stud.

[9] Elwyn G, Kreuwel I, Durand MA, Sivell S, Joseph-Williams N, Evans R (2011). How to develop web-based decision support interventions for patients: a process map. Patient Educ Couns.

[10] Cooke J, Langley J, Wolstenholme D, Hampshaw S (2017). ‘Seeing’the difference: the importance of visibility and action as a mark of ‘authenticity’ in co-production. Int J Health Policy Manage.

[11] Kothari A, Wathen CN (2017). Integrated knowledge translation: Digging deeper,moving forward. J Epidemiol Community Health.

[12] Rycroft-Malone J, Burton CR, Bucknall T, Graham ID, Hutchinson AM (2016). Collaboration and co-production of knowledge in healthcare: opportunities and challenges. Int J Health Policy Manage.

[13] Better Services by Design. http://www.bsbd.org.uk/. Accessed 27 June 2020.

[14] Simons H, Mccormack B (2007). Qualitative inquiry integrating arts-based inquiry in evaluation opportunities and challenges. Qual Inq.

[15] Psallidas I, Kalomenidis I, Porcel JM, Robinson BW, Stathopoulos GT (2016). Malignant pleural effusion : from bench to bedside. Eur Respir Rev.

[16] Havelock T, Teoh R, Laws D, Gleeson F. Pleural procedures and thoracic ultrasound: British Thoracic Society pleural disease guideline 2010. Thorax. 2010;65(Suppl. 2):i61–76. 10.1136/thx.2010.137026 20696688

[17] Davies HE, Mishra EK, Kahan BC, Wrightson JM, Stanton AE, Guhan A (2012). Effect of an indwelling pleural catheter vs chest tube and talc pleurodesis for relieving dyspnea in patients with malignant pleural effusion: the TIME2 randomized controlled trial. J Am Med Assoc.

[18] Fleishman SB, Sugarbaker DJ, Richards WG, Demmy T, Kohman L, Daniel TM (2005). Phase III intergroup study of talc Poudrage vs talc slurry sclerosis for malignant pleural effusion. Chest.

[19] Bhatnagar R, Maskell N. The modern diagnosis and management of pleural effusions. Br Med J. 2015;351:h4520. 10.1136/bmj.h4520 26350935

[20] ‘My Pleural Effusion Journey’. https://mypleuraleffusionjourney.com/. Accessed 17 July 2020.

[21] Clarke J, Waring J, Timmons S (2018). The challenge of inclusive coproduction: the importance of situated rituals and emotional inclusivity in the coproduction of health research projects. Soc Policy Adm.

[22] Bec R, Langley J, Wolstenholme D (2018). 741. Designing in Health: Developing Shared Knowledge through (Un-)Prototyping.

[23] Point of Care Foundation.https://www.pointofcarefoundation.org.uk/resource/experience-based-co-design-ebcd-toolkit/. Accessed 27 Jan 2020.

